# Effect of Yeast Probiotic on Growth, Antioxidant Enzyme Activities and Malondialdehyde Concentration of Broiler Chickens

**DOI:** 10.3390/antiox2040326

**Published:** 2013-11-06

**Authors:** Tagang Aluwong, Mohammed Kawu, Moshood Raji, Tavershima Dzenda, Felix Govwang, Victor Sinkalu, Joseph Ayo

**Affiliations:** 1 Department of Physiology, Faculty of Veterinary Medicine, Ahmadu Bello University, Zaria 810006, Nigeria; E-Mails: mukawu@yahoo.com (M.K.); drdzenda@yahoo.com (T.D.); felixgovwang@yahoo.com (F.G.); sinkmayo@yahoo.com (V.S.); joayo94@yahoo.com (J.A.); 2 Department of Microbiology, Faculty of Veterinary Medicine, Ahmadu Bello University, Zaria 810006, Nigeria; E-Mail: rajmash2002@yahoo.com

**Keywords:** body weight, broilers, enzyme activity, malondialdehyde, probiotic, reactive oxygen species

## Abstract

The aim of the study was to determine the effect of yeast probiotic on body weight, and the activities of anti-oxidant enzymes: superoxide dismutase (SOD), catalase (CAT) and glutathione peroxidase (GPx), and malondialdehyde (MDA) concentration of broiler chickens. The experiment was carried out on hybrid Hubbard broiler chickens (*n* = 200). Two-hundred day-old chicks were randomly selected and distributed into four groups of 50 day-old chicks each: Control, C, and treatment groups comprising *T*_1_, *T*_2_ and *T*_3_ administered with 0.25 mL, 0.5 mL and 1.0 mL yeast probiotic, respectively. Chicks were fed a commercial starter diet for the first 28 days of age, followed by pelleted finisher diet from 29 to 42 days. Chickens in T_1_ had a significantly (*p* < 0.01) higher body weight at 4th week of age when compared with the control. SOD activity in all treatment groups was not significantly (*p* > 0.05) different when compared with the control. GPx activity was significantly (*p* < 0.01) higher in *T*_1_, when compared with the control. GPx activity in *T*_2_ was higher (*p* < 0.01) when compared with the control. There was no significant (*p* > 0.05) difference in MDA level in all the treatment groups. In conclusion, administering yeast probiotic supplement increased body weight and enhanced serum anti-oxidant enzyme activities of broiler chickens.

## 1. Introduction

Probiotics are viable microbial feed supplements, which stimulate the growth and health as well as modify the ecology of the intestine in a beneficial manner for the host [1,2]. They have been successfully used to improve the health of poultry and to achieve better production [3,4]. Feeding of *Saccharomyces cerevisiae* to broiler chickens enhances growth performance in a dose-dependent manner [5]. Nutrition plays a huge role in maintaining the pro-oxidant-antioxidant balance [6]. An excessive production of reactive species (RS) causes an imbalance in the pro-oxidants/anti-oxidants system, and any imbalance in favor of the pro-oxidants potentially leading to damage is termed oxidative stress [7]. Recently, an adapted concept of oxidative stress (OxS) was advanced by Jones [8], which states that OxS causes disruption of redox signaling and control, emphasizing the impact of the redox ratio as vital tools for the quantification of oxidative stress. Broiler chickens are exposed to natural or induced stressors, some of which induce reactive oxygen species (ROS) generation, which may promote morphological and/or physiological malfunctions. Presently, much attention has been focused on new approaches of anti-oxidant therapy by providing anti-oxidant enzymes. All aerobic organisms possess antioxidant enzymes capable of preventing membrane cell damage, enzyme inactivation and nucleic acid alterations. Main anti-oxidant enzymes that constitute the first line of anti-oxidant enzymatic defenses include superoxide dismutase (SOD) [EC.1.15.1.1], catalase (CAT) [EC.1.11.1.6] and glutathione peroxidase (GPx) [EC.1.11.1.9]. SOD catalyzes dismutation of superoxide radicals to hydrogen-peroxide and oxygen; CAT catalyzes the breakdown of hydrogen-peroxide to water and molecular oxygen. GPx is a selenium-dependent enzyme, which decomposes peroxides using the peptide glutathione (GSH) as its co-substrate [9]. Enzymatic ROS scavengers are catalase and peroxidases that lower the steady-state concentration of H_2_O_2_, a precursor of potent radical species. Thus, the cytotoxic potential of H_2_O_2_ is predominantly a function of intracellular catalase and peroxidase activities that scavenge H_2_O_2_ and concentration of free ions of transition metals that promote generation of OH from H_2_O_2_. ROS have been considered as the major mediators of oxygen cytotoxicity and as important messengers, stimulating cell division and demonstrating cellular signaling effects [10]. A concentration of MDA in tissues and urine are generally used as a biomarker for radical-induced damage and endogenous lipid peroxidation [11,12].

*Saccharomyces cerevisiae* cells respond to oxidative stress by altering their transcriptional program in a complex manner [13,14,15]. Zhang *et al*. [5] reported improve oxidative stability in male broiler chickens supplemented with *S. cerevisiae*. However, there is paucity of information on anti-oxidant enzyme activity of yeast probiotic used as feed supplement in broiler chickens using *in vivo* assays. Based on this background, the aim of the study was to determine the effect of yeast probiotic on growth and activity of main anti-oxidant enzymes and malondialdehyde concentration in broiler chickens. We hypothesized that different concentrations of yeast probiotic administered orally to broiler chickens enhance growth, anti-oxidant enzyme activities and maintain tissue malondialdehyde concentration.

## 2. Experimental Section

### 2.1. Experimental Design and Animal Management

The experiment was carried out on hybrid Hubbard broiler chickens. Two hundred day-old chicks were randomly selected and distributed into four groups of 50 day-old chicks each: Control, C; *T*_1_ 0.25 mL, *T*_2_ 0.5 mL and *T*_3_ 1.0 mL yeast probiotic treatment groups. Birds were housed in an environmentally controlled poultry house with floor covered with wood shavings. The shavings were kept dry throughout the experimental period by routine replacement of the spoiled litter. Ambient temperature and relative humidity were 28.9 °C and 92%, respectively. Chicks were administered with Newcastle disease vaccine (NDV-intra-ocular), Infectious bursal disease vaccine (IBDV), and Newcastle disease vaccine (NDV-La Sota) on day 3, 14, and 21, respectively. The yeast probiotic was orally administered to each chick in the experimental groups through the beak, at the concentration of 0.5 mL/L of drinking water daily, using Unitract^®^ 1 mL Tuberculin (TB) syringes (Clinicare, Mumbai, India). This is a single culture probiotic, containing *Saccharomyces cerevisiae* at 4.125 × 10^6^ CFU per 100 mL. The quantity of probiotic administered to the first treatment group (*T*_1_) 0.25 mL) was ½ the standard concentration and unreconstituted in drinking water. Chicks were fed a commercial broiler starter diet for the first 28 days of age, and pelleted finisher diet from 29 to 42 days of age. Birds had 15 h of light and 9 h of darkness throughout the experimental period. The ingredients and nutrient levels of experimental diets, and the chemical composition of diet carried out by proximate analysis are shown in Table 1 and Table 2, respectively. Feed and water were provided *ad libitum*. Body weight (BW), feed conversion ratio (FCR), and feed intake (FI) were recorded on a weekly basis for comparative evaluation and interaction effects of all treatment groups. Mortality was observed and recorded on daily basis. After 42 days of feeding, blood was collected from 15 randomly selected broiler chickens from each group through the jugular or brachial vein into polythene tubes after 5 h of fasting. Blood (*i.e.*, serum samples) was used for the analysis of antioxidant enzymes and MDA.

**Table 1:** Ingredients and nutrient levels of experimental diets.

Ingredients	Starter	Finisher	Starter Nutrient levels		Finisher Nutrient levels	
Maize	N/S	N/S	ME, Kcal/Kg	2800	ME, Kcal/kg	2900
Soybean	N/S	N/S	CP, %	20	CP, %	19
Palm kernel cake	N/S	N/S	Ca, %	1.0	Ca, %	1.0
Wheat offal	N/S	N/S	Available P, %	0.45	Available P, %	0.40
Fish meal	N/S	N/S	CF, %	9	CF, %	10
Blood meal	N/S	N/S	Fat, %	10	Fat, %	10
Bone meal	N/S	N/S				
Oyster shell	N/S	N/S				
l-Lysine	N/S	N/S				
l-Methionine	N/S	N/S				
Vit/Min premix	N/S	N/S				
Salt	N/S	N/S				
Total	N/S	N/S				

N.B. Nutrient levels are values declared by manufacturer on the feed labels. N/S = Not stated.

**Table 2:** Proximate analysis of starter and finisher feeds.

Ingredient (%)	Starter	Finisher
Dry matter	93.87	94.86
Crude protein	23.69	21.20
Crude fibre	4.87	6.14
Oil	6.04	5.31
Ash	7.86	7.88
NFE	57.54	59.47

### 2.2. Body Weight Measurement

Birds were weighed individually at weekly intervals using Mettler Toledo^®^ digital precision weighing balance with a sensitivity of 0.01 g (Model MT-500D).

### 2.3. Carcass and Organ Weight Measurement

Ten fasted broiler chickens from a total of 50 birds per treatment group were randomly selected, and each was reweighed just before slaughtering. Immediately after stunning in an electrobath, the birds were slaughtered by severing the jugular vein. After evisceration, the abdominal fat was collected and weighed. Eviscerated carcasses and organs were individually weighed using a Mettler Toledo^®^ digital precision weighing balance. The main meat parts (*i.e.*, thighs and drumsticks), wings, shanks and other organs were weighed for each carcass to determine the individual part to carcass ratio. That is, organ yield was calculated using the formula: organ weight/carcass weight × 10.

### 2.4. Enzymatic Measurements

The activity of GPx [EC.1.11.1.9] was measured using the Paglia and Valentine [16] spectrophotometry method based on the Northwest Life Science Specialties (NWLSS™) Glutathione peroxidase assay kits protocol NWK-GPX01. The activity of SOD [EC.1.15.1.1] was assessed using the NWLSS™ Superoxide dismutase activity assay, which provided a simple, rate method for determining SOD activity. This method is based on monitoring the auto-oxidation rate of haematoxylin as originally described by Martin Jr., *et al*. [17], with modifications to increase robustness and reliability. Briefly, in the presence of SOD enzyme at specific assay pH (pH 7.4), the rate of auto-oxidation was inhibited and the percentage of inhibition is linearly proportional to the amount of SOD present within a specific range. Sample SOD activity was determined by measuring ratios of auto-oxidation rates in the presence and absence of the sample and expressed as traditional McCord-Fridovich “cytochrome c” units. The activity of CAT [EC.1.11.1.6] was determined spectrophotomerically according to the method of Beers and Sizer [18], with modifications to increase robustness and convenience using the NWLSS™ Catalase activity assay kits protocol NWK-CAT01. MDA level was analyzed using the standard method of NWLSS™ MDA assay kits NWK-MDA01. This was based on the reaction of MDA with thiobarbituric acid (TBA); forming an MDA-TBA_2_ adduct that absorbed strongly at 532 nm [19]. Enzyme activity was expressed as units per millilitre (U/mL) of the sample.

### 2.5. Statistical Analysis

GraphPad Windows 4.03 (GraphPad Software, San Diego, CA, USA) was used for statistical analyses and data obtained were expressed as the mean ± standard error of the mean (Mean ± SEM). Data were analyzed using repeated measures ANOVA. Turkey’s post hoc test was used to compare all treatment groups with the control and between treated groups. Values of *p* < 0.05 were considered significant.

## 3. Results and Discussion

### 3.1. Body Weight, Feed Intake and Feed Conversion Ratio of Broiler Chickens

Results are shown in Table 3, Table 4 and Table 5. Body weights from week 1 and 2 were highly significant (*p* < 0.001) in *T*_2_ group but significantly (*p* < 0.05) different in the same *T*_2_ group in week 3 when compared with the control group, respectively. In addition, there was a significant (*p* < 0.001) difference in body weight in *T*_2_ and *T*_3_ in week 2 when *T*_2_ and *T*_3_ were compared with *T*_1_ and *T*_2_, respectively. In week 3, significance differences (*p* < 0.01) and (*p* < 0.05) in body weights were observed between *T*_2_ and *T*_1_, and between *T*_3_ and *T*_2_, respectively. There were significant (*p* < 0.01) differences in body weight between *T*_2_ and *T*_1_ and between *T*_3_ and *T*_1_, respectively in week 4. However, in week 5, a significant (*p* < 0.05) difference in body weight only exists between *T*_3_ and *T*_1_. There were highly significant (*p* < 0.001) differences between *T*_1_ and *T*_2_; *T*_1_ and *T*_3_. In the sixth week of the experiment, there was no significant difference (*p* > 0.05) in body weight between the treated groups and the control. Overall, the mean body weight obtained in *T*_1_ (1269 ± 31.17 g) in the sixth week was higher when compared with other experimental groups and the control. Feed intake in week 3 decreased (*p* < 0.05) in *T*_2_ and *T*_3_ when compared with the control group. However, there was a significant reduction (*p* < 0.01) in FI between *T*_1_ and *T*_2_, and between *T*_1_ and *T*_3_, respectively. In the fourth week, FI decreased (*p* < 0.001) significantly in *T*_2_ and *T*_3_ when compared between treated groups, and with the control. A similar trend of decreased (*p* < 0.001) in FI was observed in week 5 and 6, respectively. A significant improvement in FCR was recorded in the first (*T*_1_) treatment group. In the sixth week of age, there was significant (*p* < 0.05) decrease in feed conversion ratio of broiler chickens in *T*_1_ when compared with the control. The feed conversion ratio of broiler chickens in the sixth week of the experiment for control, *T*_1_, *T*_2_, and *T*_3_ were 0.97, 0.49, 0.64, and 0.53, respectively. Feed conversion efficiency did not differ (*p* > 0.05) in all the treatment groups in the 1st week of the experiment. During the six weeks of the experimental period, the percentage of mortality in each group was 24, 12, 20 and 16% for control, *T*_1_, *T*_2_, and *T*_3_, respectively.

The present study revealed that probiotic supplementation had an impact on the body weight of broiler chickens from the fourth week of life. This may be explained as one of the yeast probiotic beneficial effects of promoting a healthy gastrointestinal tract environment by nourishing the enterocytes, improving ileal mucosal development and reinforcing mucosal barrier function through maintaining epithelial integrity. This finding is similar with the result obtained by Zhang *et al*. [5], who reported improve growth rate of male broiler chickens supplemented with *S. cerevisiae*. In addition, this finding agrees with the results obtained by several authors [20,21] in studies with broiler chickens. The highest body weight was recorded in the experimental group administered with the lowest probiotic concentration (*T*_1_ 0.25 mL). This very concentration was directly administered to the birds daily without reconstitution in drinking water. However, *T*_2_ could not cause an increase in body weight as seen in *T*_3_. This probably may be associated with the dose effect in *T*_3_, which is higher than the *T*_1_ dose. This result demonstrated that single strain *Saccharomyces cerevisiae* (Antox^®^ probiotic) supplementation has a more positive effect on body weight when administered in lower concentrations, without being reconstituted in drinking water as prescribed by the manufacturer (Montajat Veterinary Pharmaceutical Co. Ltd., Dammam, Saudi Arabia). Santin *et al*. [22] showed that the cell walls of *Saccharomyces cerevisiae* improve nutrient absorption from the intestinal mucosa and suggested that this factor may be responsible for the improvement in performance of broiler chickens supplemented with *S. cerevisiae*. A probiotic acts by reducing the feed conversion ratio, resulting in an increase in daily live weight gain [23], which is achieved through a natural physiological way and improvement of digestion by balancing the resident gut microflora. The differences in final body weight in the sixth week may be associated with differences in feed intake.

**Table 3:** Effect of supplemental yeast probiotic on body weight of broilers. Significance difference is indicated by single and double asterisk as: * *p* < 0.05 *vs*. control, ** *p* < 0.01 *vs*. control, *** *p* < 0.001 *vs*. control; C, control group (without probiotic); *T*_1_, first treatment group; *T*_2_, second treatment group; *T*_3_, third treatment group. The data are presented as Mean ± SEM, (*n* = 30). Significance difference at *p* < 0.05.

Week	C	*T*_1_ 0.25 mL	*T*_2_ 0.5 mL	*T*_3_ 1.0 mL	*p*-values
1	90.97 ± 1.98	91.63 ± 1.14	67.63 ± 2.45 ***	89.77 ± 1.61 ***	*p* < 0.0001
2	185.4 ± 5.25	199 ± 3.84	147.9 ± 6.34 ***	185.4 ± 5.29 ***	*p* < 0.0001
3	402.7 ± 12.55	425.3 ± 10.13	355.4 ± 16.57 *	407.9 ± 12.48 *	0.0015
4	647.4 ± 17.29	733.6 ± 13.93 **	611.6 ± 23.92 ***	544.7 ± 18.15 **	*p* < 0.0001
5	886.0 ± 30.77	926.5 ± 18.59	862.1 ± 38.19	804 ± 32.50 *	0.0322
6	1157 ± 49.55	1269 ± 31.17	1141 ± 43.29	1143 ± 46.48	0.1307

**Table 4:** Effects of dietary yeast probiotic supplement on feed intake of broilers. Significance difference is indicated by single and double asterisk as: * *p* < 0.05 *vs*. control, ** *p* < 0.01 *vs*. 0.5, *** *p* < 0.001 *vs*. control. C, control group (without probiotic); *T*_1_, first treatment group; *T*_2_, second treatment group; *T*_3_, third treatment group. The data are presented as Mean ± SEM, (*n* = 30). Significance difference at *p* < 0.05.

Week	C	*T*_1_ 0.25 mL	*T*_2_ 0.5 mL	*T*_3_ 1.0 mL	*p*-values
1	424.3 ± 68	574.3 ± 116	365.7 ± 33	557.4 ± 108	0.0395
2	1161.0 ± 91	1175.0 ± 103	1097.0 ± 97	1089.0 ± 91	*p* < 0.0001
3	2149.0 ± 222	2206.0 ± 205	1771.0 ± 139 *	1805.0 ± 137 *	*p* < 0.0001
4	2958.0 ± 133	3007.0 ± 132	2559.0 ± 107 ***	2579.0 ± 105 ***	*p* < 0.0001
5	5379.0 ± 424	5521.0 ± 343	4342.0 ± 273 ***	4377.0 ± 271 ***	*p* < 0.0001
6	7283.0 ± 116	7570.0 ± 173	5856.0 ± 65 ***	6159.0 ± 107 ***	*p* < 0.0001

**Table 5:** Effect of supplemental yeast probiotic on feed conversion ratio of broilers. C, control group (without probiotic); *T*_1_, first treatment group; *T*_2_, second treatment group; *T*_3_, third treatment group.

Week	C	*T*_1_ 0.25 mL	*T*_2_ 0.5 mL	*T*_3_ 1.0 mL
1	0.16	0.21	0.24	0.22
2	0.33	0.24	0.39	0.31
3	0.27	0.21	0.26	0.22
4	0.33	0.22	0.30	0.52
5	0.66	0.64	0.53	0.48
6	0.79	0.49	0.64	0.53

In the present study, there were significant differences in feed intake between treated birds and control birds. This may be attributed to improved digestion and absorption of nutrient in the digestive tract due to the presence of live yeast cells of *Saccharomyces cerevisiae*. These findings are consistent with a series of experimental studies, which revealed that dietary prebiotics [24] and probiotics [25] increase feed intake of broiler chickens. However, it is in contrast with the findings by [26,27] who reported that dietary additions of probiotics and organic acid preparations, respectively, did not affect feed intake of broiler chickens. Improvement in feed intake by dietary probiotic and prebiotic supplementation often resulted in improved growth performance. Furthermore, significant differences in feed intake existing between treated birds and the control group may be partly responsible for the variation in growth performance. Nevertheless, the birds supplemented with the lowest concentration of the probiotic were heavier than the birds in other treatment groups with the control (C) inclusive. A significant improvement in feed conversion efficiency was recorded in the first experimental group (*T*_1_) treated with the probiotic. FCR values for broiler chickens treated with 0.25 mL of dietary *Saccharomyces cerevisiae* probiotic possessed the highest body weight at the 6th week. These results are in agreement with the findings of Jin *et al*. [28] who reported that although a significant improvement in feed conversion ratio was observed in probiotic-supplemented broilers chickens, the results were inconsistent. Probiotics act by reducing the feed conversion, thereby resulting in an increase in daily live weight gain. This may be attributable to efficient ileal digestibility of nutrients. Szymczyk *et al*. [29] reported a marked reduction in feed conversion efficiency in animals fed 1.5% conjugated linoleic acid-supplemented diet relative to control. In addition, Bansal *et al*. [23] reported significant and better weekly feed conversion efficiency on probiotic supplementation in the diet of commercial broiler chicks.

### 3.2. Carcass and Organ Weights of Broiler Chickens

Weights and yields for carcasses and some organs of broiler chickens are presented in Table 3. Birds in *T*_1_ experimental group tended to be heavier than birds in *T*_2_ and *T*_3_ experimental groups. However, they were much heavier (*p* < 0.01) when compared with the control group. Weights of the thighs and drum sticks in *T*_1_ were higher (*p* < 0.05) when compared with the control group, but the yield percentages were not. Abdominal fat weight was significantly low (*p* < 0.001) in all probiotic-supplemented groups when compared with control group. This was consistent with the decreased yield percentages in all the probiotic supplemented groups. There was no significant difference (*p* > 0.05) in the weights of the heart and intestine in all the treated groups. In addition, there was no significant difference (*p* > 0.05) in the weights of the gizzards, liver, spleen, gall bladder and lungs. The differences in carcass weights of broiler chickens may originate from differences in feed conversion ratio and feed intake. In the present study, the feed conversion ratio was low in *T*_1_ probiotic group (results not included) and this same group recorded the highest body weight in the xith week of the experimental period. These results further corroborated the recent findings that probiotic act by reducing the feed conversion ratio, thereby resulting in an increase in daily live weight gain [23]. The yeast probiotic in *T*_1_ was more effective in elevating the live weight of broiler chickens followed by *T*_3_ group. This may be explained as the improved digestion and absorption of nutrients in the digestive tract of broiler chickens by the live yeast cells of *Saccharomyces cerevisiae*. Abdominal fat represents the main fat deposition in broiler chickens and it seems to be directly related to total carcass fat [30,31]. Excess accumulation of abdominal fat means both processing and waste problems, but also indicates inefficient energy use [32]. In the present study, significant differences were observed in abdominal fat in probiotic-supplemented groups. There was overt decreased in weight of abdominal fat in all the probiotic-supplemented groups, indicating the fact that probiotics enhance efficient energy usage. This study is similar with several studies that reported lowering of abdominal fat by probiotic supplementation [24,30,32,33]. On the contrary, Bozkurt *et al*. [34] showed that dietary supplementation of prebiotics, organic acids and probiotics had no significant effect on abdominal fat pad accumulation in broiler chickens. The entire mass of the intestine in *T*_1_ group was heavier but not significant than the weights of intestine from other experimental groups. This result disagrees with the findings by Alcicek *et al*. [35], who reported that dietary supplementation of probiotics lowered the weight of the small intestine. In addition, there was no significant effect of the probiotic on the weights of organs like the liver, gizzard and lungs. In some previous findings, dietary prebiotic and probiotic supplementation, respectively, did not increase the liver weights of broiler chickens [28,36]. Results of probiotic effect on the carcass and organ weights of broilers are shown below (Table 6).

**Table 6:** Effect of supplemental yeast probiotic on carcass and organ weights of broilers. Significance difference is indicated by single, double and triple asterisk as: * *p* < 0.05 *vs*. control, ** *p* < 0.01 *vs*. control, *** *p* < 0.001 *vs*. control; C, control group (without probiotic); *T*_1_, first treatment group; *T*_2_, second treatment group; *T*_3_, third treatment group. The data are presented as Mean ± SEM, (*n* = 10). Significance difference at *p* < 0.05.

Parameters	C	*T*_1_ 0.25 mL	*T*_2_ 0.5 mL	*T*_3_ 1.0 mL	*p*-values
Live weight (g)	1382.0 ± 37.95	1678.0 ± 64.34 **	1482.0 ± 34.69	1515.0 ± 56.08	0.0034
Carcass weight	903.5 ± 35.47	1094.0 ± 46.68 **	987.5 ± 26.82	996.7 ± 31.77	0.0096
%	65.38	65.20	66.63	65.79	
Thigh (g)	270.0 ± 8.65	318.6 ± 14.49 *	298.9 ± 8.11	303.8 ± 10.91	0.0424
%	29.88	29.12	30.27	30.48	
Drum stick (g)	153.9 ± 4.68	178.5 ± 7.73 *	171.5 ± 5.55	170.7 ± 3.56	0.0368
%	17.03	16.32	17.37	17.13	
Abdominal fat	9.10 ± 0.18	6.60 ± 0.16 ***	7.60 ± 0.16 ***	7.10 ± 0.23 ***	*p* < 0.0001
%	1.01	0.60	0.77	0.71	
Gizzard (g)	45.00 ± 4.79	57.50 ± 2.32	54.80 ± 2.34	56.30 ± 3.89	0.1050
%	4.98	5.26	5.55	5.65	
Liver (g)	44.50 ± 1.92	45.80 ± 1.98	47.10 ± 1.96	50.00 ± 1.76	0.1455
%	4.93	4.19	4.77	5.02	
Heart (g)	9.40 ± 0.45	10.40 ± 0.52	9.40 ± 0.31	10.90 ± 0.32	0.0376
%	1.04	0.95	0.95	1.09	
Lungs (g)	10.70 ± 0.42	11.80 ± 0.49	10.80 ± 0.51	11.60 ± 0.43	0.2633
%	1.18	1.08	1.09	1.16	
Intestine (g)	151.7 ± 8.23	177.6 ± 7.64	157.3 ± 4.64	168.0 ± 7.11	0.0590
%	16.79	16.23	15.93	16.86	

### 3.3. Anti-Oxidant Enzyme Activities and Malondialdehyde Concentration of Broiler Chickens

**Figure 1:**
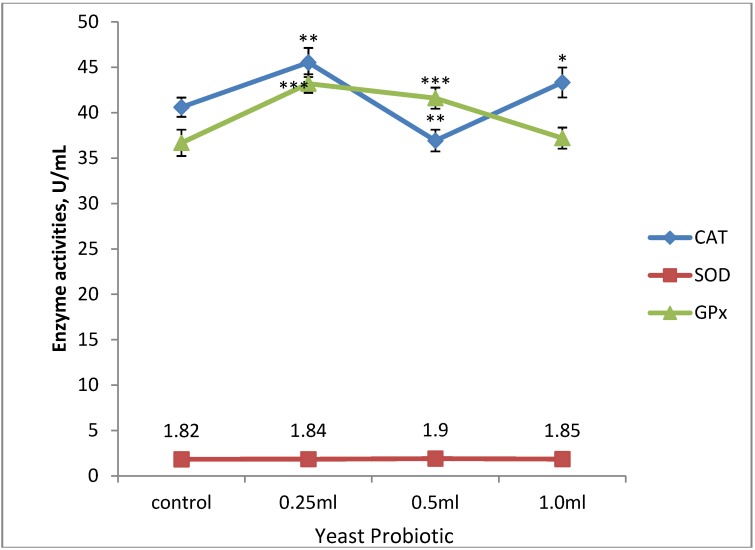
Effect of supplemental yeast probiotic on serum antioxidant enzyme activities of broiler chickens. Significance difference is indicated by single, double and triple asterisk as: * *p* < 0.05 *vs*. 1.0, ** *p* < 0.01 *vs*. 0.5, *** *p* < 0.001 *vs*. control. Data are presented as Mean ± SEM, (*n* = 15).

Results of serum anti-oxidant enzyme activities are shown in Figure 1. CAT activity was significantly higher (45.53 ± 1.60 U/mL; *p* < 0.01) in *T*_1_ when compared with *T*_2_. In addition, there was significant (*p* < 0.05) CAT activity in *T*_3_ when compared with *T*_2_. SOD activity in all the treatment groups was not significantly (*p* > 0.05) different when compared with the control and treatment groups. GPx activity was greater (43.20 ± 1.02 U/mL; *p* < 0.001) in *T*_1_ when compared with *T*_3_ and control group.

In addition, a more significant (*p* < 0.01) difference in GPx activity exists between *T*_2_ and the control. When *T*_1_ was compared with *T*_3_ and *T*_2_ with *T*_3_, a significant (*p* < 0.05) difference was observed between these treatment groups. There was significant (*p* < 0.05) difference in MDA concentration in *T*_1_ when compared with *T*_3_.

**Figure 2:**
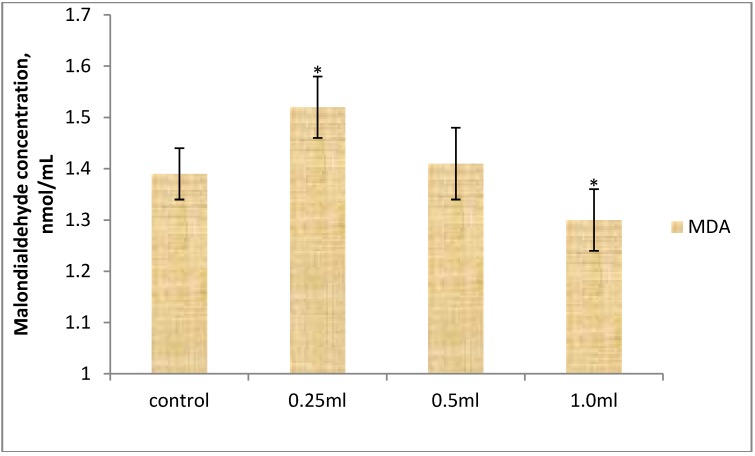
Effect of supplemental yeast probiotic on serum malondialdehyde concentration of broiler chickens. Significance difference is indicated by single asterisk as: * *p* < 0.05 *vs*. 1.0. Data are presented as Mean ± SEM, (*n* = 15).

Anti-oxidant enzymes are most effective when acting synergistically with one another or with other components of the anti-oxidant barrier of the organism when their activity remains balanced. It has been shown that nutrition plays a vital role in maintaining the pro-oxidant-antioxidant balance [6]. In the present study, there was increased in both catalase and glutathione peroxidase activities. The increased in activity of these antioxidant enzymes may be attributed to the age, colonization resistance, and susceptibility to environmental pathogens of the birds. This is in agreement with the work of numerous scholars [37,38] who reported that an increased in the activity of catalase in the blood is caused by environmental burdens to which birds are exposed to during their growth. In addition, most importantly growth processes in early life is characterized with the generation of ROS through cellular division and apoptosis. This is because ROS are considered as the major mediators of oxygen cytotoxicity and as important messengers stimulating cell division and manifesting cellular signaling effects [10]. A similar study in turkey reported that mannanoligosaccharides a component of *S. cerevisiae* used as dietary additive stimulate the mechanisms of oxidative defense and improve the growth performance of the birds [39]. Krizkova *et al*. [40] showed that mannans from *S. cerevisiae* have antioxidative property *in vitro*. This suggests that dietary yeast can protect the gastrointestinal tract in ways other than just removing undesirable bacteria. In addition, Kogan *et al*. [41] suggested that yeast cell wall β-glucans may have antioxidant activity. The steady state of SOD activity in the probiotic-supplemented groups may reflect a significant improvement in health and oxidative status of the birds. These findings are contrary to results obtained by Milinkovic-Tur *et al*. [42], who reported increased activities of SOD and CAT in the heart muscles of broiler chickens. Catalase activity, as well as the activity of other anti-oxidant enzymes, depends on the presence of anti-oxidants in the diet. Oxidative damage develops when anti-oxidant potential is reduced and/or when factors contributing to oxidative stress increase [43,44]. The increase in GPx activity in *T*_1_ suggests greater oxidative stress. Also, GPx increased activity in *T*_2_ may be attributed to the sodium selenite fraction of the yeast (*S. cerevisiae*) probiotic, which partly enhances anti-oxidative activity. The present results further corroborate the existing knowledge on the positive effect of selenium on GPx activity in chicken erythrocytes, blood, muscles and the liver [45,46]. GPx displays its activity mainly in the cellular cytoplasm and only about ten percent of activity is displayed in the mitochondria [47]. In this manner, the safe removal of hydrogen peroxide is attained through the joint action of GPx and CAT. Malondialdehyde level endogenously reflects lipid peroxidation, which is the sequela of diminished anti-oxidant protection as ROS levels increase. MDA level was higher in *T*_1_ (Figure 2), and this is a direct reflection of GPx activity in *T*_1_. This may be attributed to the probiotic inability to confer adequate anti-oxidant protection against lipid peroxidation during the growth phase of the birds. We are unaware of any research work on *S. cerevisiae* as anti-oxidant and used to study oxidative stress during growth in broiler chickens.

## 4. Conclusions

In conclusion, to maintain the physiological grade of oxidative stress needed for a number of bio-functions like growth, broiler chickens have an integrated antioxidant defense system that is enhanced by the yeast probiotic. However, it has an inability to confer adequate anti-oxidant protection against lipid peroxidation during growth. This therefore, confirms that *S. cerevisiae* is an optimal eukaryotic model to study cellular events related to oxidative stress.

## References

[1] Breves G., Walter C., Burmeister M., Shroder B. (2000). *In vitro* studies on the effects of *Saccharomyces boulardii* and *Bacillus cereus* var. toyoi on nutrient transport in pig jejunum. J. Anim. Physiol. Anim. Nutr..

[2] Simon O., Vahjen W., Scharek L. Microorganisms as Feed Additive-Probiotics. Proceedings of the 9th International Symposium on Digestive Physiology in Pigs.

[3] O’dea E.E., Fasenko G.M., Allison G.E., Korver D.R., Tannock G.W., Guan L.L. (2006). Investigating the effects of commercial probiotics on broiler chick quality and production efficiency. Poult. Sci..

[4] Peric L., Milosevic N., Zikic D., Bjedov S., Cvetkovic D., Markov S., Mohnl M., Steiner T. (2010). Effect of probiotic and phytobiotic products on performance, gut morphology and cecal microflora of broiler chicks. Arch. Tierz..

[5] Zhang A.W., Lee B.D., Lee S.K., Lee K.W., An G.H., Song K.B., Lee C.H. (2005). Effects of yeast (*Saccharomyces cerevisiae*) cell components on growth performance, meat quality and ileal mucosa development of broiler chicks. Poult. Sci..

[6] Cowey C.B. (1986). The role of nutritional factors in the prevention of peroxidative damage to tissues. Fish Physiol. Biochem..

[7] Sies H. (1991). Oxidative Stress: Oxidants and Antioxidants.

[8] Jones D.P. (2006). Redefining oxidative stress. Antioxid. Redox Signal..

[9] Halliwell B. (2006). Reactive species and antioxidants redox biology is a fundamental theme of aerobic life. Plant Physiol..

[10] Buetler T.M., Krauskopf A., Ruegg U.T. (2004). Role of superoxide as a signalling molecule. News Physiol. Sci..

[11] Day C.P. (1996). Is necro-inflammation a prerequisite for fibrosis?. Hepatogastroenterology.

[12] Wang Y.Z., Xu C.L., An Z.H., Liu J.-X., Feng J. (2008). Effect of dietary bovine lactoferin on performance and antioxidant status of piglets. Anim. Feed Sci. Technol..

[13] Gasch A.P., Spellman P.T., Kao C.M., Carmel-Harel O., Eisen M.B., Storz G., Botstein D., Brown P.O. (2000). Genomic expression programs in the response of yeast cells to environmental changes. Mol. Biol. Cell..

[14] Ikner A., Shiozaki K. (2005). Yeast signalling pathways in the oxidative stress response. Mutat. Res..

[15] Temple M.D., Perrone G.G., Dawes I.W. (2005). Complex cellular responses to reactive species. Trends Cell Biol..

[16] Paglia D.E., Valentine W.N. (1967). Studies on the quantitative and qualitative characterization of erythrocyte glutathione peroxidase. J. Lab. Clin. Med..

[17] Martin J.P., Dailey M., Sugarman E. (1987). Negative and positive assays of superoxide dismutase based on haematoxylin auto-oxidation. Arch. Biochem. Biophys..

[18] Beers R.F., Sizer I.W. (1952). A spectrophotometric method for measuring the breakdown of hydrogen peroxide by Catalase. J. Biol. Chem..

[19] Placer Z.A., Cushman L.L., Johnson B.C. (1996). Estimation of lipid peroxidation, malondialdehyde in biochemical system. Ann. Biochem..

[20] Gohain A.K., Sapcota D. (1998). Effect of probiotics feeding on the performance of broilers. Indian J. Poult. Sci..

[21] Morales-Lopez R., Auclair E., van Immerseel F., Ducatelle R., Garcia F., Brufau J. (2010). Effects of different yeast cell wall supplements added to maize- or wheat-based diets for broiler chickens. Br. Poult. Sci..

[22] Santin E., Maiorka A., Macari M., Grecco M., Sanchez J.C., Okada T.M., Myasaka A.M. (2001). Performance and intestinal mucosa development of broiler chickens fed diets containing *Saccharomyces cerevisiae* cell wall. J. Appl. Poult. Res..

[23] Bansal G.R., Singh V.P., Sachan N. (2011). Effect of probiotic supplementation on the performance of broilers. Asian J. Anim. Sci..

[24] Safalaoh A.C.L. (2006). Body weight gain, dressing percentage, abdominal fat and serum cholesterol of broilers supplemented with a microbial preparation. Afr. J. Food Agric. Nutr. Dev..

[25] Sanchez R., Ayaya J.A. (1998). Effect of MOS on Broiler Performance under Field Conditions.

[26] Yeo J., Kyu-il K. (1997). Effect of feeding diets containing an antibiotic, a probiotic, or yucca extract on growth and intestinal urease activity in broiler chicks. Poult. Sci..

[27] Sims M.D., Sefton A.E. Comparative Effects of Mannan-Oligosaccharide and an Antibiotic Growth Promoter on Performance of Commercial Broilers. Presented at the 50th North Central Avian Disease Conference.

[28] Jin L.Z., Ho Y.W., Abdullahi N., Ali M.A., Jalaludin S. (1998). Effects of adherent Lactobacillus cultures on growth, weight of organs and intestinal microflora and volatile fatty acids in broilers. Anim. Feed Sci..

[29] Szymczyk B., Pisulewski M.P., Szczurek S. (2001). Effects of conjugated linoleic acid on growth performance, feed conversion efficiency, and subsequent carcass quality in broiler chickens. Br. J. Nutr..

[30] Santoso U., Tanaka K., Ohtani S. (1995). Effect of dried *Bacillus subtilis* culture on growth, body composition and hepatic lipogenic enzyme activity in female broiler chicks. Br. J. Nutr..

[31] Gaya L.G., Mourao G.B., Rzende F.M., Chicaronide Matos E., Filho T.M., Figueiredo L.G.G., Ferraz J.B.S., Eler J.P. (2005). Genetic trends of abdominal fat content in a male broiler chicken line. Genet. Mol. Res..

[32] Homma H., Shinohara T. (2004). Effects of probiotic *Bacillus cereus toyoi* on abdominal fat accumulation in Japanese quail (*Coturnix japonica*). Anim. Sci. J..

[33] Anjum M.L., Khan A.G., Azim A., Afzal M. (2005). Effect of dietary supplementation of multi-strain probiotic on broiler growth performance. Pak. Vet. J..

[34] Bozkurt M., Kucukyilmaz K., Cath A.U., Cinar M. Growth Performance and Carcass Yield of Broiler Chickens Given Antibiotic, Mannan Oligosaccharide and Dextran Oligosaccharide Supplemented Diets. Nutritional Biotechnology in the Feed and Food Industries, Proceedings of Alltech’s 21st Annual Symposium.

[35] Alcicek A., Bozkurt M., Cabuk M. (2004). The effects of a mixture of herbal essential oil, an organic acid or a probiotic on broiler performance. S. Afr. J. Anim. Sci..

[36] Bozkurt M., Kucukyilmaz K., Cath A.U., Cinar M. (2009). The effect of single or combined dietary supplementation of prebiotics, organic acid and probiotics on performance and slaughter characteristics of broilers. S. Afr. J. Anim. Sci..

[37] Gutowicz M., Cholojczyk M., Pyrzanowska R.M., Cholojczyk M., Pyrzanowska J., Widy-Tyszkiewicz E., Baranczyk-Kuzma A. (2008). Effect of curcumin on antioxidant and detoxification mechanisms in the livers of aging rats. Med. Weter..

[38] Lecewicz A., Jankowski J., Zdunczyk Z., Juskiewicz J. (2008). Selected factors stimulating the development of some gastrointestinal parts in turkeys. Med. Weter..

[39] Ognik K., Krauze M. (2012). Dietary supplementation of mannanoligosaccharides to turkey hens on their growth performance and antioxidant status in blood. S. Afr. J. Anim. Sci..

[40] Krizkova L., Durackova Z., Sandula J., Sasinkova V., Krajcovic J. (2001). Antioxidative and antimutagenic activity of yeast cell wall mannans *in vitro*. Mutat. Res..

[41] Kogan G., Pajtinka M., Babincova M., Miadokova E., Rauko P., Slamenova D., Korolenko T.A. (2008). Yesat cell wall polysaccharides as antioxidants and antimutagens: Can they fight cancer?. Neoplasma.

[42] Milinkovic-Tur S., Aladrovic J., Ljubic B.B., Poljicak-Milas N. (2009). Age-related antioxidant enzyme activities and lipid peroxidation in heart muscles of broiler chickens fed with supplementary organic selenium. Vet. Arhiv..

[43] Ibrahim W., Lee U.S., Yen H.C., Siclair D.K., Chow C.K. (2000). Antioxidant and oxidative stress status in tissues of manganese superoxide dismutase transgenic mice. Free Radic. Biol. Med..

[44] Milinkovic-Tur S., Stojevic Z., Pirsljin J., Zdelar-Tuk M., Poljicak-Milas N., Beer Ljubic B., Gradinski-Vrbanac B. (2007). Effect of fasting and refeeding on the antioxidant system in cockerels and pullets. Acta Vet. Hung..

[45] Kuricova S., Boldizarova K., Geresakova L., Bobcek R., Levkut M., Leng L. (2003). Chicken selenium status when fed a diet supplemented with Se-yeast. Acta Vet. Brno.

[46] Pirsljin J., Milinkovic-Tur S., Beer Ljubic B., Zdelar-Tuk M. (2008). The effect of organic selenium supplementation on the antioxidative characteristics and lipid peroxidation of chicken blood during fattening and after fasting. Vet. Arhiv..

[47] Halliwell B., Gutteridge J.M.C. (1999). Free Radical Biology and Medicine.

